# Content validity of the EQ-5D-5L with skin irritation and self-confidence bolt-ons in patients with atopic dermatitis: a qualitative think-aloud study

**DOI:** 10.1007/s11136-023-03519-6

**Published:** 2023-10-03

**Authors:** Eszter Szlávicz, Ákos Szabó, Ágnes Kinyó, Anita Szeiffert, Tamás Bancsók, Valentin Brodszky, Rolland Gyulai, Fanni Rencz

**Affiliations:** 1 Department of Dermatology, Venereology and Oncodermatology, Medical School, University of Pécs, 1 Akác Street, Pécs, Hungary; 2 Department of Health Policy, Corvinus University of Budapest, Budapest, Hungary; 3 Károly Rácz Doctoral School of Clinical Medicine, Semmelweis University, Budapest, Hungary

**Keywords:** EQ-5D-5L, Atopic dermatitis, Skin irritation, Self-confidence, Bolt-on, Content validity

## Abstract

**Objectives:**

Two bolt-on dimensions (skin irritation, self-confidence) have been developed for the EQ-5D-5L to improve its content validity and responsiveness in psoriasis. However, the two bolt-ons are not strictly psoriasis-specific and are potentially relevant in other skin conditions. This study aims to explore the content validity of the EQ-5D-5L with two bolt-ons in patients with atopic dermatitis (AD).

**Methods:**

In 2021–2022, qualitative, semi-structured interviews were conducted with 20 adult AD patients at a university dermatology clinic in Hungary. We aimed for a heterogeneous sample in terms of age, gender, education and disease severity. Patients completed the EQ-5D-5L with two bolt-ons using a think-aloud protocol. Probing questions were posed to investigate item relevance, potential conceptual overlaps, missing concepts and the appropriateness of the recall period. Interview transcripts were subjected to thematic analysis.

**Results:**

The EQ-5D-5L with the two bolt-ons covered the most important aspects of health-related quality of life in AD patients. Most patients found both the skin irritation and self-confidence bolt-ons relevant. Fifteen potential missing concepts were identified, but only two (social relationships, judgement by others) were identified by more than one patient. A smaller conceptual overlap was found between the skin irritation and pain/discomfort dimensions in 7 patients (35%). Half the patients expressed a preference for a recall period of 1 week rather than of ‘today’.

**Conclusions:**

The EQ-5D-5L with skin irritation and self-confidence bolt-ons showed good relevance, comprehensiveness and comprehensibility in patients with AD. However, in terms of comprehensiveness, social relationships and judgement by others (stigma) may be missing from the questionnaire.

**Supplementary Information:**

The online version contains supplementary material available at 10.1007/s11136-023-03519-6.

## Introduction

The EQ-5D is the most frequently applied health-related quality of life (HRQoL) measure to obtain quality-adjusted life year (QALY) estimates for cost-utility analyses worldwide [1–3]. Despite its brevity, it shows good validity, discriminatory power and responsiveness in a broad range of health conditions and settings [4]. However, its content validity may be limited in some specific populations as certain aspects of HRQoL are not adequately covered by the EQ-5D [5, 6]. The addition of bolt-on dimensions to the descriptive system is an approach to improve content validity of the EQ-5D [7]. Some candidate bolt-on dimensions include cognition, fatigue, sleep, social relationships, breathing problems, speech, vision and hearing [8, 9].

EQ-5D is commonly used in the context of skin conditions, including one of the most frequent chronic dermatological diseases, psoriasis [10–12]. To improve its content validity, two bolt-ons (skin irritation and self-confidence) were developed for the five-level EQ-5D (EQ-5D-5L) in psoriasis patients, which are collectively referred to as ‘EQ-PSO’ [13]. While all bolt-ons are considered modifications of the EQ-5D, the skin irritation and self-confidence bolt-ons are ‘beta status’ instruments. They are not yet finalized (‘approved’) products, but their development has reached a more advanced stage. Moreover, while other bolt-ons are typically available in only a very limited number of languages, these two are available in almost 50 different languages for users [14]. The two bolt-ons have exhibited good validity and responsiveness in psoriasis [13, 15, 16]. They are not strictly psoriasis-specific in terms of their content and may have relevance in other chronic dermatological conditions. However, there may be differences in the characteristics and patterns of symptoms, particularly itching, among various skin conditions. These differences can include variations in intensity, timing, localization, trigger and alleviating factors [17, 18]. As a result, it becomes essential to assess the content validity of these bolt-ons in other relevant dermatological conditions (e.g. atopic dermatitis, hidradenitis suppurativa, autoimmune bullous skin diseases, chronic urticaria). Furthermore, in other chronic skin diseases, additional or alternative examples may be necessary to improve comprehensiveness of the skin irritation bolt-on. For example, skin burning or stinging could be relevant examples. Similarly, the level of self-confidence problems may also depend on the localization and visibility of skin symptoms, which differs across skin conditions. Self-confidence issues are likely more pronounced when symptoms appear on visible body areas [19].

Atopic dermatitis (AD) is a chronic inflammatory skin disease that is the most common type of eczema and a leading cause of global disease burden from skin diseases [20]. It affects up to 12% of the adult population and shows an increasing prevalence globally [21]. Patients with AD typically experience dryness and intense itching of the skin, potentially leading to erythema, skin injuries, pain, infections and sensitization to allergens [22]. The most commonly affected body areas are the head, neck and hands. Patients frequently report disturbances in their usual activities and adverse mental health consequences, such as mood disorders, anxiety and concentration problems due to itching and sleeping problems [23–25]. These symptoms may also deeply affect social life and self-esteem [26, 27]. The EQ-5D-5L has been widely used in AD patients, demonstrating improved measurement properties compared to the EQ-5D-3L [28–31]. However, similarly to psoriasis, the EQ-5D-5L might not fully capture all relevant aspects of HRQoL in AD.

The objective of this study is therefore to investigate the content validity (relevance, comprehensiveness and comprehensibility) of the EQ-5D-5L with the skin irritation (itching) and self-confidence bolt-ons in adult AD patients.

## Methods

### Participants, study design

Reporting of this study follows the Standards for Reporting Qualitative Research (SRQR) checklist [32]. Data collection was approved by the Hungarian Medical Research Council (no. IV/2374-1 /2021/EKU). Between May 2021 and February 2022, semi-structured, face-to-face interviews were conducted with Hungarian patients diagnosed with AD. Patients were recruited at the Department of Dermatology, Venereology and Oncodermatology of the University of Pécs in Hungary. Any AD patient treated at the university dermatology clinic that met the inclusion criteria was eligible to participate in the study. The aim was to achieve a balanced sample in terms of age, gender, education, type of treatment and disease severity. The inclusion criteria for this study were as follows: (1) aged 18 years or above; (2) diagnosed with AD by a dermatologist; (3) cognitive ability to understand the questions in Hungarian; and (4) written informed consent. Patients were recruited to the study until data saturation was reached (i.e. when no new themes emerged in the last three interviews) [33].

### Interviews

All interviews were performed by the first author (E.S.), who is a dermatologist with a clinical PhD and previous experience in interviewing and treating AD patients. The interviews were performed in a quiet room at the university’s dermatology clinic library. This study was part of a longer interview session that also included two condition-specific questionnaires (DLQI, Skindex-16), with the EQ-5D-5L and bolt-ons positioned first. Qualitative findings about the other two instruments will be reported elsewhere.

A topic guide was developed by the research team building on a similar study in psoriasis patients in Hungary (Online Resource 1) [14]. The topic guide was pilot-tested and refined with feedback from two AD patients. In the first part of the interview, participants were asked about the important aspects of their lives affected by AD. This section was followed by completing the EQ-5D-5L, the two bolt-ons and EQ VAS using a concurrent think-aloud protocol [34]. Patients verbalized their thoughts when filling in the questionnaire. Probing questions were used to investigate three areas of content validity (relevance, comprehensiveness, comprehensibility) as recommended by the COnsensus-based Standards for the selection of health Measurement Instruments (COSMIN) standards [35]. Patients were asked about the meaning and relevance of the dimensions, potential overlaps between concepts, missing concepts related to HRQoL, appropriateness of the response levels, wording and recall period. In the last section of the interview, patients were asked to complete a short background questionnaire comprising questions about their sociodemographic and clinical characteristics, such as disease duration, comorbidities and treatments. Disease severity was assessed by a dermatologist using the SCORing Atopic Dermatitis (SCORAD) [36, 37].

### EQ-5D-5L and bolt-ons

The EQ-5D-5L consists of two parts, a descriptive system and a vertical visual analogue scale (EQ VAS) with endpoints of 0 (the worst health you can imagine) and 100 (the best health you can imagine) [38]. The descriptive system covers five dimensions of HRQoL: mobility, self-care, usual activities, pain/discomfort and anxiety/depression. In the present study, the skin irritation and self-confidence bolt-ons were positioned before the EQ VAS [13]. On each of the five core dimensions as well as the two bolt-ons, respondents are asked to assess their HRQoL using five response options (‘no problems’ to ‘extreme problems’/’unable to’). The skin irritation dimension measures the level of itching experienced by the patient, with response options ranging from ‘no itching’ to ‘extreme itching’.

### Data analysis

All interviews were anonymously audio-recorded, and the recordings were transcribed verbatim. A thematic analysis was performed by two researchers (a dermatologist who also conducted the interviews and a health outcomes researcher with a PhD in dermatology) [39]. The data analysis involved multiple stages. In the first stage, the two coders read all the transcripts, and the coder who was not an interviewer, also listened to the recordings. In the second step, the coders identified the main codes in the transcripts following the themes from the topic guide. In the third stage, both researchers coded the first three interviews and discussed any disagreements. The coding framework was also discussed with other members of the research team. The remaining interviews were coded by one of the coders and verified by the other. Trustworthiness of the analysis was assured by involving multiple researchers with different relevant academic backgrounds in developing the main themes and coding, and involving multiple members of the research team in case of any disagreements between the coders. To allow summarizing the qualitative data, an extraction matrix was developed in Microsoft Excel 2016. Categories and sub-categories were created and the matrix was populated with quotations from patients. To report the findings, we selected and translated the most expressive quotations from the interviews to English.

## Results

### Sample characteristics

Overall, 23 participants were enrolled in the study. Three participants did not show up, resulting in a final sample of 20 patients. The sample was heterogeneous in terms of age, gender, education and disease severity. Data saturation was achieved after the sixteenth interview. A detailed description of the sample characteristics can be found in Table 1.

**Table 1:** Characteristics of the study population

Variables of the population	Median (range) or n (%)
Age (years)	25 (18–45)
Gender	
Female	12 (60%)
Male	8 (40%)
Education	
Secondary	11 (55%)
College/university	9 (45%)
Disease duration (years)	23 (5–44)
Comorbidities	
Food allergy	9 (45%)
Allergic rhinitis	8 (40%)
Bronchial asthma	6 (30%)
Anxiety	5 (25%)
Allergy to animal hair	3 (15%)
Chronic sinusitis	2 (10%)
Metal allergy	2 (10%)
Antiphospholipid syndrome	1 (5%)
Deep vein thrombosis	1 (5%)
Arrhythmia	1 (5%)
Immune thrombocytopenic purpure	1 (5%)
Latex allergy	1 (5%)
Vitiligo	1 (5%)
Facial involvement	12 (60%)
Hand involvement	11 (55%)
Current therapy	
Topical	10 (50%)
Photo/systemic non-biological	3 (15%)
Biological (≤ 6 weeks)	2 (10%)
Biological (> 6 weeks)	5 (25%)
EQ VAS (0–100)	81.5 (25–98)
Disease severity (SCORAD)	
Score	32.6 (0–82.2)
Clear (0–9.9)	3 (15%)
Mild (10.0–28.9)	7 (35%)
Moderate (29.0–48.9)	5 (25%)
Severe (49.0–103)	5 (25%)

*SCORAD* SCORing Atopic Dermatitis

### Impact of AD on patients’ lives

A total of 36 important aspects of patients’ lives were identified through open-ended questions related to the medical history, treatments and quality of life issues. Patient responses were organized into subcategories, which were then condensed into main categories. Finally, five categories were obtained: (1) symptoms and disease course, (2) treatment difficulties, (3) limitations in daily activities, (4) mental health problems and (5) intra- and interpersonal relations of the individual (Table 2). Among these aspects, itch was the most frequently reported problem. Except for one participant, all patients experienced this symptom and its consequences; for example, how it affected their daily activities, such as work or studying, sports and sleeping. Several physical symptoms, including erythema, bleeding, wound development, head and neck involvement, were also considered important problems, mentioned by more than half of the patients. Another, often discussed aspect was the impact of AD on appearance, social interactions with family or colleagues and their romantic relationships. Psychological disturbances, especially shame (mentioned by 11 participants, 55%), were also commonly raised. Four patients mentioned low self-worth and two patients reported the lack of self-confidence due to their AD.

**Table 2:** Important aspects of lives in patients with atopic dermatitis

Main categories	Subcategory	n	%	EQ-5D-5L dimension
Symptoms and disease course	Itch	19	95	PD, SI
	Exacerbation/progression	18	90	PD
	Head and neck symptoms	15	75	CO
	Bleeding/wounds	11	55	PD, SI
	Erythema	11	55	AD
	Skin irritation/burning sensation	10	50	PD, SI
	Circadian/seasonal fluctuation	9	45	PD
	Dryness	8	40	SI
	Risk of infection	8	40	
	Co-morbidities	7	35	
	Pain	7	35	PD, SI
	Skin scaling	7	35	PD
	Discomfort	2	10	PD, SI
Treatment difficulties	Therapy resistance	16	80	
	Time/cost and other resources	12	60	
	Side effects	9	45	
	Waiting for treatment	4	20	
Limitations in activities	Self-care	13	65	SC
	Diet	6	30	
	Work/studies	5	25	UA
	Sport	5	25	UA
	Sleeping	4	20	SI
	Swimming pool activities	4	20	UA
Mental health problems	Shame	11	55	AD
	Anxiety	5	25	AD
	Frustration	5	25	
	Depression	4	20	AD
	Stress	4	20	AD, PD
Intra- and interpersonal relations of the individual	Appearance	15	75	AD, CO
	Acceptance of disease	12	60	CO
	Stigmatization	11	55	AD, CO
	Social interactions (family, work, school)	8	40	AD, CO
	Peer support	8	40	CO
	Relationship problems	4	20	AD, CO
	Self-worth	4	20	CO
	Lack of self-confidence	2	10	CO

EQ-5D-5L dimensions were matched with subcategories based on the life aspects emerged during the think aloud processes of the patients

The n and % values relate to number of patients that spontaneously described aspect of life as important

*AD* anxiety/depression, *CO* self-confidence, *HRQoL* health-related quality of life, *PD* pain/discomfort, *SC* self-care, *SI* skin irritation, *UA* usual activities

### Relevance of the EQ-5D-5L and bolt-ons

Patients found the EQ-5D-5L and bolt-ons to be easy to complete and comprehensive. Almost all participants considered the seven dimensions relevant to their disease: *“It’s related to the disease. So, all these questions or statements reflect what I experience every day (…) I find it straightforward.”* One patient expressed uncertainty whether the questionnaire was intended to be a generic or a condition-specific instrument: *“I didn’t understand whether I should relate my responses to eczema, or just answer in general.”* Another participant highlighted the irrelevance of certain dimensions for younger patients: *“(…) mobility and self-care, although I suppose these are more for elderly patients who might have difficulties with these”.* No negative comments emerged regarding the relevance of the other dimensions. Moreover, patients gave positive feedback regarding the importance of the skin irritation and self-confidence bolt-ons: *“Q: Skin irritation. A: This is the best question so far (…) Q: Why would you say this is the best question? A: Because this is perhaps the most important problem with this whole disease. This is the main problem.”* and *“I would not cover myself up, but the disease is annoying, thus I don’t have much self-confidence”.*

### Order of dimensions

The patients agreed that the order of dimensions is acceptable; however, three participants mentioned that the order of questions is not important. Some patients highlighted that *“it is well established, dealing first with usual activities, and subsequently turning towards the mental, emotional entities”*. There were suggestions to rearrange the dimensions by grouping related concepts together, such as *“anxiety, depression and self-confidence might be together in one group, consecutively”* and *“putting skin irritation after pain and discomfort, maybe more interrelated; personally, I would connect them more closely”.*

### Dimension content

Overall, the majority of participants interpreted the seven dimensions as intended. In case of the mobility dimension, 7 (35%) patients mentioned difficulties related to sports in addition to walking, and two participants also referred to mobility problems related to skin irritation: "*Well, I think this condition doesn’t really affect mobility much. It is a bit of a disadvantage when doing sports and sweating, which can be very irritating”*. On the other hand, there was a misinterpretation observed for the self-care dimension. Specifically, 12 patients (60%), especially those with moderate-to-severe AD, understood problems with washing or dressing not as difficulties performing these activities, but rather as the skin irritation caused by certain clothes, cosmetics and topical treatments (e.g. ointments) leaving a stain on their clothes or causing skin irritation. One patient summarized these problems as follows: *“I have to be very careful when selecting soaps, lotions or ointments due to their potential to cause irritation. Sometimes only clear water can help. Choosing clothing that comes into direct contact with my skin, is extremely burdensome, as everything makes it itchy.”*

Regarding skin irritation, most patients interpreted the dimension according to their perceived level of itch: *“When my symptoms are severe then I also experience very strong itching. During these times, I tend to scratch my skin until it bleeds.”* Two participants mentioned sleep disturbances in relation to the skin irritation dimension. While most patients associated skin irritation with itch, one patient mentioned that it also includes dryness: *“Q: And what does this irritation mean? A: Mostly itching, but in the mornings I also experience dryness when I wake up, then I usually apply a moisturizer. So, it’s these two things.”* The self-confidence dimension was interpreted as how individuals perceive themselves (e.g. being a valued person) and how they believe others may judge them: “*I often feel ugly because I have eczema on my face as well; even when I’m fully clothed, people can still see it.”*

### Ranking of dimensions

The majority of participants considered skin irritation as the most relevant dimension (n = 9, 45%), followed by pain/discomfort and anxiety/depression (n = 5, 35%) (Table 3). None of the patients indicated mobility as being the most relevant dimension. On the other hand, mobility was most frequently mentioned as the least relevant dimension (n = 8, 40%). Importantly, skin irritation, self-confidence and pain/discomfort were not considered the least relevant dimensions by any of the patients.

**Table 3:** Most and least relevant dimensions

Dimension	Most relevant		Least relevant	
	n	%	n	%
Mobility	0	0	8	40
Self-care	2	10	4	20
Usual activities	1	5	1	5
Pain/discomfort	8	40	0	0
Anxiety/depression	5	25	2	10
Skin irritation	9	45	0	0
Self-confidence	3	15	0	0

Note that some patients ranked more than one dimension as the most or least relevant. 7 patients did not rank any dimension as the least relevant

### Missing concepts

Altogether 15 missing concepts were mentioned by 16 (80%) patients. These were classified into three groups: (1) AD-related, (2) general health-related and (3) other aspects related to well-being (Table 4). The most frequently reported missing concepts were problems with social relationships (n = 5) and judgement by others (n = 4). Other aspects, such as appearance, sexual life, sleeping and sports, were raised by only one patient each.

**Table 4:** Missing concepts from the EQ-5D-5L with two bolt-ons

Type of concepts	Concepts	Patients mentioning each concept		Patients mentioning ‘type of concept’	
		n	%	n	%
Atopic dermatitis-related	Control of itch	1	5	5	25
	Irritation by clothing or bathing	1	5		
	Past severity of symptoms	1	5		
	Risk of infection	1	5		
	Seasonal fluctuation	1	5		
	Treatment and its efficacy	1	5		
General health-related	Diet	1	5	5	25
	Sexual life	1	5		
	Sleeping	1	5		
	Sport	1	5		
	Tension	1	5		
Other aspects	Problems with social relationships	5	25	10	50
	Judgement by others	4	20		
	Appearance	1	5		
	Cost of products	1	5		

Altogether, 16 patients mentioned at least one missing concept. Six patients reported multiple missing concepts, but none of them reported more than two different ones

### Conceptual overlaps between the dimensions

The thematic analysis revealed few overlaps between the dimensions (Table 5). Among these, the most frequent overlap was between skin irritation and pain/discomfort. Itch was described by seven patients (35%) as a form of discomfort or pain. Further, one patient pointed out an overlap between anxiety/depression and self-confidence, while another patient mentioned an overlap between anxiety/depression and pain/discomfort.

**Table 5:** Conceptual overlaps between EQ-5D-5L and bolt-on dimensions

Dimension	Overlap	n	%	Example quote
Pain/discomfort	Anxiety/depression	1	5	P11: Sometimes I also feel very bad and if I am going through a slightly more stressful period my wounds hurt a lot and I feel terrible. I tend to show symptoms of depression
Skin irritation	Pain/discomfort	7	35	P20: It was a terrible itch, but I wouldn’t call it as pain. I might not assess it accurately and it can actually be called pain
				P04: It's not a type of pain you typically associate with someone being in pain, but it was such a bad sensation. So that the inflamed skin burns and itches
Self-confidence	Anxiety/depression	1	5	P19: I had a problem with my self-confidence, and I still have to some extent. I believe anxiety also plays a role in how others perceive me

### Suggested changes

Altogether 9 (45%) patients suggested at least one change to one or more of the seven dimensions (Table 6). Suggestions for changes were made to all dimensions except self-confidence. Some patients recommended to better elaborate the content of certain dimensions (mobility, self-care and skin irritation) or splitting the composite dimensions (i.e. pain/discomfort and anxiety/depression) into two questions. One patient suggested dividing the usual activities dimension into work/housework and family/leisure activities. No specific wording changes were suggested, except for one patient who requested a clearer definition of discomfort (i.e., clarify whether it refers to physical or mental discomfort). Finally, it was also suggested to expand the self-care dimension to include eating.

**Table 6:** Suggested changes to the EQ-5D-5L and bolt-ons

Themes	n	%	Example quote
Mobility			
Further define mobility	2	10	P01: When it comes to mobility, it does matter if it is a change in location or position. Maybe I do not have problem with walking but when I have to bend down or lift something then there is a problem
Self-care			
Add eating	1	5	P01: Covers only cleaning and dressing (…) so can I eat alone. Do I have a problem with that or do I need help
Further define washing and dressing	1	5	P04: So, there could be more about getting dressed, and about washing, yes, so the process of bathing could be discussed
Usual activities			
Split into work-housework and family-leisure activities	1	5	P04: It would be possible to separate work and learning, they are not the same activity for me. (…) I would put it separately, let's say work-housework and family-leisure (…) I also do sports and I would emphasise it a little more because it prevents [the disease] people from doing sports
Pain/discomfort			
Split it into 2 questions	3	15	P06: I would not associate mild pain and discomfort because I can have large pain and feel good even though it is strange, why shouldn't it be? On the other hand, without pain I can have major discomfort
Further define discomfort	1	5	P03: I would only paraphrase discomfort (…) that they mean exactly mentally, or rather physically, such as, for example, when a person has a fever and feels bad
Anxiety/depression			
Split into 2 questions	3	15	P19: I would not necessarily put anxiety and depression on the same level, because I think that someone can have major anxiety without having any symptoms of depression
Skin irritation			
Further define itch	1	5	P010: There could have been one or two more questions about the nature of the itching. (…) to know what provokes it or on which body part does it occur or what makes it go away?
Self-confidence	0	0	No changes suggested

### Response levels

Most participants were satisfied with the five levels used across the dimensions and found them to be easily understandable and well distinguishable. However, three patients (15%) had concerns about the response levels. One patient recommended using a 1–10 scale to reflect the extent of bothersome symptoms. This patient also found it challenging to distinguish between the response levels: *“I do not think that there will be major differences between the five levels. Apparently, there is a distinction between the first and last levels, but not between the single steps”.* Another participant would have preferred a questionnaire with numbered response levels instead of verbal descriptions. Three patients interpreted the response levels of the skin irritation bolt-on as a frequency scale rather than severity: *“I would mark "mild itching", because the irritation is still there, just not as visibly on my skin. Q: When would you mark "moderate"? A: I don't know, maybe if this irritation occurred much more frequently.”*

### EQ VAS

The findings on the interpretations of EQ VAS are reported in Online Resource 2.

### Recall period

Only one patient used an incorrect recall period. Six participants stated that they would have provided the same responses with a different recall period. Only two patients agreed to apply “today” as the recall period, the other patients preferred a longer recall period due to the daily fluctuations in their disease symptoms. Two patients mentioned itching and none mentioned self-confidence in their responses: *“I think ‘today’ is not appropriate to describe these problems. My back is much itchier than it was yesterday.”* Most patients (n = 10, 50%) reported that a 1-week recall period would be the most informative, and six patients (30%) proposed an even longer, such as 2 weeks or 1–3 months. Two patients were uncertain about the ideal recall period.

## Discussion

Over 10 years ago, a skin irritation and a self-confidence bolt-on were developed for the EQ-5D-5L in psoriasis [13]. However, these dimensions are not exclusive to psoriasis, as they may be relevant in other chronic skin conditions, and even extend beyond dermatology. For example, they could be valuable in systemic conditions that cause itching (e.g. chronic kidney failure, certain liver diseases, some forms of cancer) or in any illness potentially causing self-confidence problems (e.g. generalized anxiety disorder). Previous lines of research have used the skin irritation/itching bolt-on (with multiple wordings) in adult patients with psoriasis and burn injuries [13, 15, 16, 40, 41], while the self-confidence bolt-on has been used in psoriasis, myasthenia gravis and investigating the effect of the COVID-19 pandemic on the general population [13, 15, 16, 42, 43]. To our knowledge, this is the first study to use the skin irritation and self-confidence bolt-ons in patients with AD.

Our findings provide strong support for the use of the skin irritation bolt-on in AD patients. The majority of patients identified skin irritation as their main problem, and this dimension was also very commonly ranked as the most relevant in the ranking exercise. Compared to skin irritation, the relevance of self-confidence was not as direct, it was still clearly highlighted. Before completing the questionnaire, six participants reported self-worth or self-confidence as important areas of HRQoL in AD. Furthermore, several patients mentioned additional aspects of HRQoL that may be considered as consequences or related difficulties (e.g. social relationships, judgement by others). To gain a deeper insight into this question, we reviewed the patients’ interpretations of the self-confidence descriptor. Few patients associated this dimension with skills and achievements in specific activities, such as work and sports. Two patients discussed discrimination in general without connecting it to the dimension to AD. On the other hand, most patients answered the question considering their skin disease, emphasizing the relevance of self-confidence in AD. Importantly, no respondents considered either skin irritation or self-confidence as the least relevant aspect of HRQoL.

There is limited evidence available regarding the number of bolt-ons that can be attached to the EQ-5D. In measuring population health, several bolt-ons can be added to the EQ-5D. However, when the aim is to improve content validity and responsiveness in a specific condition, studies typically limit the number of bolt-ons to one or two. Examples include conditions such as hemophilia, burn, cataract, visual disorders and hearing impairments [41, 44–47]. The most commonly reported missing concepts in AD were judgement by others and difficulties with social relationships. Recently, multiple social functioning-related bolt-ons (e.g. social relationships, social participation, community connectedness) have been proposed for the EQ-5D-5L, which could be potentially useful in AD patients [42, 48]. However, it is worth noting that during the development of the skin irritation and self-confidence bolt-ons, social relationships was initially considered but later dropped after the exploratory factor analysis [13]. There might be some overlap between the social relationships and self-confidence bolt-ons, as self-confidence problems often lead to difficulties in social relationships. Furthermore, sleep could also be a relevant candidate for a bolt-on in this population [49]. On the other hand, while additional dimensions might help to provide a more detailed overview of the patients’ HRQoL, they may also compromise the practicality and brevity of the instrument. This approach aligns well with the goal of keeping the descriptive system size amenable for valuation and reduce burden on respondents.

The EQ-5D questionnaires were primarily designed for generating utilities for cost-utility analyses of health technologies, and currently, national value sets are available for the five-dimension descriptive system in over 30 countries, including Hungary [50–52]. Bolt-ons are considered modifications of the EQ-5D and with few exceptions [11, 13, 50, 53], no value sets are available for bolt-ons. A value set was developed for the EQ-5D-5L and skin irritation and self-confidence bolt-ons using a combination of time trade-off and visual analogue scale in the UK from a general population sample; therefore, it is not specific to psoriasis [13]. Albeit, this did not follow the EQ-VT protocol [54] and the comparability of values with those obtained using national value sets is currently unknown. Currently, bolt-on value sets may be used as a sensitivity analysis for the reference case in health technology assessment submissions [55].

The thematic analysis identified potential conceptual overlaps between the dimensions, with the most remarkable overlap observed between the skin irritation and pain/discomfort dimensions, as reported by over one-third of the participants. A previous study conducted with psoriasis patients in Hungary also revealed a similar conceptual overlap [14]. While this might indicate a limitation in using this bolt-on; importantly, the meaning of discomfort could subtly differ in Hungarian compared to other languages [16], indicating a potential language-specific issue. On the other hand, the majority of patients could unambiguously differentiate between the two dimensions. Given that almost all patients found the skin irritation dimension relevant for describing their HRQoL problems associated with AD and the majority did not report a conceptual overlap with pain/discomfort, the skin irritation bolt-on seems to be a useful addition to the descriptive system of the EQ-5D-5L in this patient population.

A few limitations of our study need to be noted. Firstly, there might have been some selection bias affecting the recruitment process. All patients were recruited from a university dermatology clinic, which means that our sample consisted of individuals who had access to the best available care and treatments. Additionally, despite our best efforts, we were unable to include patients with a primary level of education in our sample. Secondly, despite the EQ-5D-5L being a generic HRQoL measure, certain words or phrases were interpreted in the context of AD. This was particularly evident for self-care, where several, mostly moderate-to-severe AD patients, misinterpreted the dimension as problems with clothing and applying certain cosmetics or treatments on their skin rather than having problems with the physical activities of washing or dressing (e.g. being unable to put on socks). Similar findings have been observed previously in psoriasis [14]. However, it is possible that this finding is mostly an artefact of the study protocol. The patients were specifically invited to these interviews due to their AD diagnosis, and at the beginning of the interviews, they were asked to discuss their HRQoL problems in relation to AD. This might have led them to overly focus on their skin condition when completing the EQ-5D-5L.

Taken together, our study established the degree of content validity of the EQ-5D-5L and the skin irritation and self-confidence bolt-ons in adult AD patients. However, in terms of comprehensiveness, social relationships and judgement by others (stigma) may be missing from the questionnaire. The potential conceptual overlap between the skin irritation and pain/discomfort and the potential misinterpretation of the self-care dimension warrant further research in different languages and contexts. Quantitative validation studies are recommended to test the measurement properties of these two and possible additional bolt-ons specifically in the context of AD.

### Supplementary Information

Below is the link to the electronic supplementary material.

Supplementary file1 (DOCX 22 KB)

## Data Availability

All data of this study are available from the corresponding author upon reasonable request.

## Code availability

N/A.
