# Supplementary material for: Content validity of the EQ-5D-5L with skin irritation and self-confidence bolt-ons in patients with atopic dermatitis: a qualitative think-aloud study
Source: Qual Life Res. 2023 Oct 3;33(1):101–11. doi: 10.1007/s11136-023-03519-6 (PMC10784357; doi:10.1007/s11136-023-03519-6)
Supplement: Supplementary file 1 — Supplementary file1 (DOCX 22 KB) [file 11136_2023_3519_MOESM1_ESM.docx]

**Online Supplementary Information**

**Title: Content validity of the EQ-5D-5L with skin irritation and self-confidence bolt-ons in patients with atopic dermatitis: a qualitative think-aloud study**

**Authors:**

**Eszter Szlávicz^1^, Ákos Szabó^2,3^, Ágnes Kinyó^1^, Anita Szeiffert^1^, Tamás Bancsók^1^, Valentin Brodszky^2^, Rolland Gyulai^1*^, Fanni Rencz^2*^**

1 – Department of Dermatology, Venereology and Oncodermatology; Medical School, University of Pécs

2 – Department of Health Policy, Corvinus University of Budapest, Hungary

3 – Károly Rácz Doctoral School of Clinical Medicine, Semmelweis University, Budapest, Hungary

* These two authors have contributed equally to this work.

Correspondence: szlavicz.eszter@pte.hu

**Online Resource 1 Topic guide**

**Interview preparations:**

Bring multiple hard copies of the information sheet, consent form, recording form, EQ-5D-5L with two bolt-ons (skin irritation, self-confidence), DLQI, Skindex-16 and background questionnaire.

Tape recorder: check if the recorder is working before each interview.

Setting: ensure the patient is at ease and the room is quiet. (Please wear a mask, unless the restrictions are relaxed.)

1. **Welcome and introduction**
   1. Welcome the patient and introduce yourself.
   2. Hand the information sheet and explain the aims of the study and data confidentiality. Make clear that primarily we are not collecting survey data on patients, rather we are testing questionnaires, we are interested in the ways they arrive at their answers. Give the patient enough time to read the information sheet and ask questions if there is any.
   3. Hand the written informed consent form and ask the patient to read and sign it if they are willing to participate.
   4. Ask permission to record the interview. Turn on the tape recorder.
2. **Experience with atopic dermatitis**

Example probes:

- How has atopic dermatitis affected your quality of life?

- Which daily activities were influenced by your atopic dermatitis?

- When did your symptoms begin and what were they like when it started?

- Over the years, how has the course of disease been for you?

- What kind of treatments have you tried earlier and what type of therapy are you currently receiving?

- How have you responded to the current therapy?

- How does the treatment affect your everyday life?

- How would you rate the severity of your current skin symptoms?

- Which areas of your skin are affected?

- Which symptom is the most bothersome?

- How do currently feel emotionally about your disease?

1. **Explanation of the think aloud method**

In this section we are interested in knowing what your thoughts are when completing some measures of health. In order to do this, I am going to ask you to think aloud as you work on the problems you are given. What I mean by ‘think aloud’ is that I want you to say out loud everything that comes to your mind to the extent possible, so we can tell what you are thinking about when you answer questions. Please know that there are no wrong answers, I am just interested in what is going through your mind. Please do not plan out what you are saying.

1. **EQ-5D-5L with bolt-ons and EQ VAS**

Hand out the EQ-5D-5L descriptive system with bolt-ons and EQ VAS to the patient, ask them to complete it and think aloud while responding to the questions. When they stop talking start to ask probing questions. Let the flow of the interview guide you, there is no need to follow the order of probes in the table below, but please make sure that you cover all topics at some point.

***EQ-5D-5L descriptive system and bolt-ons***

| PURPOSE | PROBES |
| --- | --- |
| General instructions | -How did you find the completion of the questionnaire?  -Could you mention any difficulties related to the understanding or the completion of the questionnaire? |
| Dimensions  (including the two bolt-ons) | -What does the [dimension] mean to you?  -What were you thinking about while you were answering the [dimension]? |
| Response levels | *(Not necessary for all dimensions, ask specifically for questions causing difficulties in answering or choosing between response options)*  -What was your reason for choosing this response?  -Why did you choose this response level instead of [response+1 level] |
| Wording | - Were there any words or expressions to change/modify or complement in order to facilitate understanding? If the answer is yes, what kind of modification would you suggest? |
| Content coverage | -What kind of other experiences do you have in relation to atopic dermatitis, not covered by the questionnaire?  -Is there any question that you do not consider relevant to atopic dermatitis?  -What do you think about the order of questions?  -What are the most and least relevant questions? Why? |
| Recall period | -Would you have responded differently if instead of ‘your health today’, the questionnaire had asked about your experiences over the past week?  -What do you consider the best recall period to describe your problems related to atopic dermatitis, ‘today’, ‘the past week’, ‘last months’ or something else? |

***EQ VAS***

| Scale | - How did you get to this response?  - What does it mean to you: “the best health state that you can imagine”?  - What does it mean to you: “the worst health state that you can imagine”? |
| --- | --- |

1. **Completion of condition-specific questionnaires (DLQI and Skindex-16) and comparison of the three questionnaires** - this part of the interview guide will be reported elsewhere.
2. **Background questionnaire**

Hand out the background questionnaire and ask the patient to complete it. After the completion of the background questionnaire, disease severity will assessed using the SCORing Atopic Dermatitis (SCORAD), by the dermatologist, who conducted the interview.

1. **Thank you and closing**

Summary, thank the patient for coming and ask if they have any questions. Provide a contact information, in case of any questions/concerns.

Turn off the tape recorder.

Complete the recording form.

**Online Resource 2 Interpretations of EQ VAS**

The majority of the participants (n=12, 60%) interpreted the endpoints (100: the best health you can imagine; 0: the worst health you can imagine) as referring to health in general. A typical explanation provided for ‘100’ was: *“I feel well in every way: mentally, physically and I do not feel pain whatsoever”*. Regarding the lower endpoint, several concepts were outlined, such as: (1) “*I think the worst health condition would be some kind of incurable, chronic disease”* (2) *“I imagine a hospitalized, dying patient”* (3) *“If one were not self-sufficient, or probably unable to move freely or reliant on the assistance of others;* *this would be the worst thing I can imagine”.* Other patients (n=7, 35%) interpreted the EQ VAS as a condition-specific scale; however, this was more frequent among patients with more severe AD (SCORAD>30). When interpreting ‘0’ on the EQ VAS, patients considered their symptoms (especially itch, wounds and bleeding) and difficulties they face in their social relationships due to their appearance: (1)*“My whole body would be red, inflamed, wounded and my skin is extremely scaling, maybe I scratch a body part until it bleeds and it [AD] is all over my skin” and* (2)*“In the past, when my skin was very inflamed, I thought it could get any worse than that (…) If the skin is unattractive, one doesn’t feel like meeting other people”*. These patients generally agreed that the best imaginable health state would be asymptomatic. One patient was uncertain in the interpretation (‘100’ was answered as generic, ‘0’ as condition-specific). Moreover, two participants initially mixed up the endpoints; however, later they noticed the error and corrected their responses.
